# Preferred place of death and end-of-life care for adult cancer patients in Iran: A cross-sectional study

**DOI:** 10.3389/fonc.2022.911397

**Published:** 2022-08-03

**Authors:** Armin Fereidouni, Mahmood Salesi, Maryam Rassouli, Fariba Hosseinzadegan, Mohammad Javid, Maryam Karami, Maryam Elahikhah, Salman Barasteh

**Affiliations:** ^1^ Quran and Hadith Research Center, Marine Medicine Research Center, Baqiyatallah University of Medical Sciences, Tehran, Iran; ^2^ Department of Operating Room Technology, School of Nursing and Midwifery, Shiraz University of Medical Sciences, Shiraz, Iran; ^3^ Chemical Injuries Research Center, Systems Biology and Poisonings Institute, Baqiyatallah University of Medical Sciences, Tehran, Iran; ^4^ Cancer Research Center, Shahid Beheshti University of Medical Sciences, Tehran, Iran; ^5^ School of Nursing and Midwifery, Urmia University of Medical Sciences, Urmia, Iran; ^6^ Students Research Committee, Baqiyatallah University of Medical Sciences, Tehran, Iran; ^7^ School of Nursing & Midwifery, Shahid Beheshti University of Medical Sciences, Tehran, Iran; ^8^ Health Management Research Center, Baqiyatallah University of Medical Sciences, Tehran, Iran; ^9^ Nursing Faculty, Baqiyatallah University of Medical Sciences, Tehran, Iran

**Keywords:** preferred place of death, preferred place of care, palliative care, end of life, cancer, hospice, Iran

## Abstract

**Background:**

More than 50,000 deaths in terms of cancer occur annually in Iranian hospitals. Determining the preferred place of end-of-life care and death for cancer patients in Iran is a quality marker for good end-of-life care and good death. The purpose of this study was to determine the preferred place of end-of-life care and death in cancer patients.

**Method:**

In 2021, the current descriptive cross-sectional investigation was carried out. Using the convenience sample approach, patients were chosen from three Tehran referral hospitals (the capital of Iran). A researcher-made questionnaire with three parts for demographic data, clinical features, and two questions on the choice of the desired location for end-of-life care and the death of cancer patients served as the data collecting instrument. Data were analyzed using SPSS software version 18. The relationship between the two variables preferred place for end-of-life care and death and other variables was investigated using chi-square, Fisher exact test, and multiple logistic regression.

**Result:**

The mean age of patients participating in the study was 50.21 ± 13.91. Three hundred ninety (69.6%) of the patients chose home, and 170 (30.4%) patients chose the hospital as the preferred place of end-of-life care. Choosing the home as a preferred place for end-of-life care had a significant relationship with type of care (OR = .613 [95% CI: 0.383–0.982], P = .042), level of education (OR = 2.61 [95% CI: 1.29–5.24], P = 0.007), type of cancer (OR = 1.70 [1.01–2.89], P = .049), and income level (*Mediate*: (OR: 3.27 (1.49, 7.14), P = .003) and *Low*: (OR: 3.38 (1.52–7.52), P = .003). Also, 415 (75.2%) patients chose home and 137 (24.8%) patients chose hospital as their preferred place of death. Choosing the home as a preferred place of death had a significant relationship with marriage (OR = 1.62 [95% CI: 1.02–2.57], P = .039) and time to diagnostic disease less than 6 months (OR = 1.62 [95% CI: 0.265–0.765], P = .002).

**Conclusion:**

The findings of the current research indicate that the majority of cancer patients selected their homes as the preferred location for end-of-life care and final disposition. Researchers advise paying more attention to patients’ wishes near the end of life in light of the findings of the current study. This will be achieved by strengthening the home care system using creating appropriate infrastructure, insurance coverage, designing executive instructions, and integration of palliative care in home care services.

## Introduction

Cancer is the second leading cause of death in the world and the third leading cause of death in Iran (1–3). According to the World Health Organization, cancer caused the deaths of 10 million people in 2020 (1 in 6 deaths) with a mean age of 72 years worldwide (4). According to GLOBALCAN statistics, more than 110,000 new cases of cancer were detected in Iran in 2018, and by 2030, this figure is projected to rise to 156,000 (5).. Iran has documented 55,785 cancer-related fatalities so far this year (1). In general, only 39% of countries report access to primary healthcare and 40% of countries report access to palliative care in community care and home care (6). Weakness in access to palliative care and end-of-life care is more serious in cancer patients. However, only 14% of cancer patients get the end-of-life care they need (out of the 34% that need it) (7). Despite these figures, the state of end-of-life care in Iran is only partially quantified. Recently, in terms of the increasing incidence of cancer and the decline in the quality of end-of-life care of these patients, this type of service has received more attention from health policymakers (8).

Dying and caring for patients’ preferred place in the last days of life are considered a quality marker to have good end-of-life care worldwide (9). Many of these patients in the final stages of life attach great importance to the preferred place for end-of-life care (PPOEOLC) and preferred place of death (PPOD), and this place will have a significant impact on their quality of life and death and care (10, 11). The terms “preferred place of death” (PPOD) and “preferred place for end-of-life care” (PPOEOLC) relate to people’s preferences for where they would want to pass away or receive care in their last days, respectively (8). Awareness of patients’ preferences about PPOEOLC and PPOD is essential for end-of-life palliative care planning (11). Besides, meeting these personal preferences is one of the ultimate criteria for success in palliative care (12). Therefore, understanding the PPOEOLC and PPOD is the first step to ensuring adequate resources for patients. The significance of this topic is shown by the many preferred surveys conducted to calculate the PPOEOLC and PPOD in the United Kingdom (9), the United States (13), European nations (14), and other countries (15). Regardless of cultural or national distinctions, the majority of cancer patients have selected their home as their PPOEOLC and PPOD (11, 12, 16). A systematic review study by Fereidouni et al. (3) in 2021 and a study by Brogaard et al. in 2013 (12) also showed that more than half of cancer patients preferred home as the PPOD and end-of-life care.

However, the most common actual place of care and death for cancer patients in different countries is the hospital (15, 17, 18). In Iran, 60% of deaths occur in hospitals (18). The reported rate of achieving a PPOD in patients varies from 49% to 88% in western countries to 66% in south Africa (16). The effect of societal and cultural factors, sociodemographic factors, clinical characteristics, and patients’ access to different palliative and psychiatric care is responsible for the variation in these data (19, 20). According to the conceptual framework created by Gomez and Higginson, the environment, the individual, and the illness all have an impact on where a person passes away. Sociodemographic details and the patient’s choices for the location of death are examples of personal considerations. Environmental factors can be attributed to healthcare inputs (home care, hospital bed availability, and hospital admissions), social support (living arrangements, patient’s social support network, and caregiver coping), and macro-social factors (historical trends, health care policy, and cultural factors) (21).

Other factors influencing the choice of PPOEOLC and PPOD include insufficient government support for palliative care as a dimension of universal health coverage, difficulty accessing drugs and inadequate training in drug use, lack of proper education, and limited financial resources in this context (22, 23). Studies have mainly examined the demographic and patients ‘clinical characteristics affecting PPOEOLC and PPOD using multiple logistic models (24–26).

Iranians are mostly Shiite Muslims (5). Death is seen as a rebirth in Iranian-Islamic culture, and each person’s death time is determined by divine destiny (27, 28). There is a distinction between a good death and a poor death in this society (28). Islam places a high value on death, as shown by the fact that the word “death” appears 84 times in the Muslim holy book, the Quran (27). Discussions about PPOC and PPOD are sensitive issues that are difficult to address without patient preparation, because they cause anxiety in the patient (11). For this reason, very few studies were conducted in Islamic countries on the PPOEOLC and PPOD. Finding a suitable and preferential place for end-of-life care and death to implement effective policies and planning based on the preferences of cancer patients is essential to providing more favorable palliative interventions. It also helps properly distribute resources to care units, such as hospitals, homes, or intermediate centers (hospices, long-term care centers) (3).

Despite all these advantages and how crucial it is to treat the PPOEOLC and PPOD issues, Iran’s health system has so far paid little attention to them. As a result, the goal of the current research was to identify PPOEOLC and PPOD in Iranian cancer patients.

## Materials and methods

### Study design

This cross-sectional descriptive study was conducted from October to November 2021. The study population was hospitalized cancer patients and referred to the outpatient department of three referral hospitals in Tehran (the capital of Iran).

### Study population

After learning the research’s goals and completing a written informed permission form, patients who satisfied the inclusion criteria joined the trial *via* convenient ways. Inclusion requirements include having received a medically confirmed diagnosis of cancer, being above the age of 18, being able to read and write Persian, being in adequate physical condition to complete the questionnaire, and not having cognitive issues such as Alzheimer’s or dementia. The required sample size was obtained based on the study of Alsirafy et al. (29) which was P = 0.28, and 345 people were obtained using the formula n = Z^2^ P (1-P)/d^2^ with 95% confidence level and (d) = 0.05; the sample size was calculated to be 370 people with design effect equal to 1.5.


$$\text{n}=\frac{Z_{1-\alpha /2}^{2}P\left(1-P\right)}{d^{2}}$$

### Data collection tool

The data collection tool included a researcher-made questionnaire consisting of three sections:

The first part includes the patient’s demographic information including age, gender, level of education, marital status, number of children, employment status, monthly income, and race; the second part includes patients’ clinical characteristics including type of care (inpatient/outpatient), type of cancer (gastrointestinal, breast, blood, other), insurance coverage (Social Security Insurance funded by the Social Security Organization, Armed Forces Insurance funded by the Ministry of Defense and Armed Forces Logistics, etc.) and time to diagnostic disease (less than 6 months, more than 6 months); and the third part includes two questions related to the PPOEOLC (home/hospital) and choosing PPOD (home/hospital).

The questionnaire’s face validity was assessed using two quantitative and qualitative techniques. By concentrating on respondents’ cognitive process while completing the scale, a cognitive interview is undertaken to determine the cause of inaccuracy in the scale (30). Ten cancer patients with diverse economic, social, and education levels were interviewed. They were requested to rate the legibility, clarity, and structure of the items, ease of comprehension, item difficulty, confusing words, item classification, ease of responding, language forms, and wording. Subsequently, the modifications were applied in the primary questionnaire. The impact score of each question was calculated to quantify face validity. For each item, the Likert scale was divided into five parts: I completely agree (score 5), I agree (score 4), I have no opinion (score 3), I disagree (score 2), and I completely disagree (score 1). Then a questionnaire was given to 10 specialists (three oncologists, five nursing professors, one palliative specialist, and one psychologist) to determine the validity. Then the impact score for each item of the questionnaire is calculated by the method (importance × frequency = impact score). If the impact score is greater than 1.5, the item is suitable (31). The impact score for both questions was more than 1.5.

“At the end of life, some individuals choose to be cared for at home, while others prefer to be cared for in a hospital,” was one of two questions connected to the PPOEOLC and the PPOD. Where would you rather get treatment as you near death? and “At the end of their lives, some individuals choose to pass away at home while others choose to pass away in a hospital. Where would you rather pass away?”

### Data analysis

After data collection, the collected data were analyzed by SPSS software version 18. Descriptive analyses including frequency and percentage were used for qualitative data, and mean and standard deviation were used for normal quantitative data. The two primary variables, the PPOEOLC and the PPOD, together with demographic factors and clinical features of the subjects were examined using chi-square and Fisher exact tests. The threshold for statistical significance was set at P 0.05. Finally, significant variables were included in the model through multiple logistic regression with the Wald backward method.

The effect of individual explanatory variables on the outcome variable was measured using the adjusted odds ratio (AOR) with a 95% confidence interval (CI).

### Ethical consideration

Permission for this study was approved by the ethics committee of Baqiyatallah University of Medical Sciences with the code of ethics (IR.BMSU.REC.1399.42). Participants were assured of anonymity and confidentiality of the information obtained.

## Result

### Demographic and clinical characteristics of the patients

The mean age of 564 patients participating in the study was 50.21 ± 13.91. Thus, 276 (48.9%) were aged 40–60 years. Furthermore, 189 (33.5%) patients were men and 375 (66.5%) were women. The number of 190 (33.8%) had elementary education, 274 (48.5%) had high school education, and 100 (17.8%) had academic education. Regarding the kind of cancer, there were 144 (28.5%) cases of gastrointestinal, 219 (43.4%) cases of breast, 55 (11.7%) cases of blood, and 83 (16.4%) cases of other cancers. In these individuals, the median time to diagnostic disease cancer was 24.83 26.18 months. Three hundred seventy-eight persons (70%) and 162 (30%) of the total population had cancer for more than 6 months, respectively (**Table 1** shows the demographic and clinical characteristics of 564 cancer patients).

**Table 1:** Demographic and clinical characteristics of the 564 cancer patients.

Variable	Categories	n (%)
**Type of care**	Outpatient	435 (77.1%)
	Inpatient	129 (22.9%)
**Sex**	Male	189 (33.5%)
	Female	375 (66.5%)
**Age (year)**	Mean ± SD*	50.21 ± 13.91
	18–40	154 (27.3%)
	40–60	276 (48.9%)
	>60	134 (23.8%)
**Marital status**	Single	126(22.4%)
	Married	437(77.6%)
**Number of children**	**Mean ± SD**	2.55 ± 1.88
	0	67 (12.2%)
	1-3	366 (66.7%)
	≥4	116 (21.1%)
**Education**	Elementary	186 (33.8%)
	High school	267 (48.5%)
	Academic	98 (17.8%)
**Job**	Employed	126 (22.6%)
	Unemployed	431 (77.4%)
**Ethnicity**	Fars	264 (47.1%)
	Lor	52 (9.3%)
	Tork	151 (26.9%)
	Kord	78 (13.9%)
	Other	16 (2.9%)
**Income**	High	42 (7.6%)
	Mediate	306 (55.4%)
	Low	204 (37%)
**Type of cancer**	Gastrointestinal	144 (28.5%)
	Breast	219 (43.4%)
	Blood	59 (11.7%)
	Other	83 (16.4%)
**Insurance**	Tamin ejtemaee	214 (38.2%)
	Military	203 (36.3%)
	Other	143 (25.5%)
**Time to diagnostic disease (month)**	Mean ± *SD	24.83 ± 26.18
	≤6	162 (30%)
	>6	378 (70%)
**Preferred place of care**	Hospital	170 (30.4%)
	Home	390 (69.6%)
**Preferred place of death**	Hospital	137 (24.8%)
	Home	415 (75.2%)

*SD, standard deviation.

### Preferred place for end-of-life care

**Figure 1:**
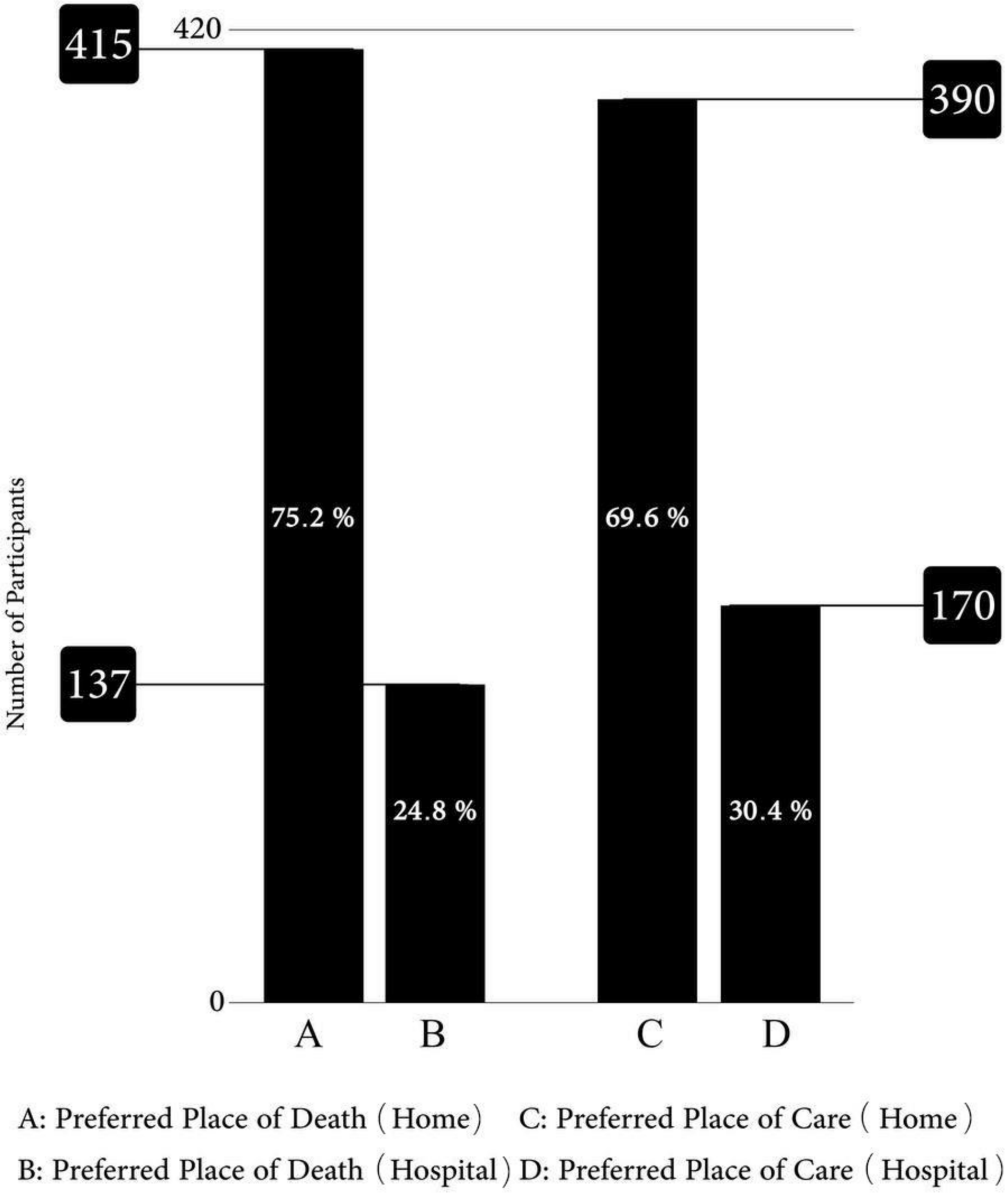
Preferred place of care and death of patients with cancer disease.

Three hundred ninety (69.6%) patients chose home and 170 (30.4%) patients chose the hospital as their preferred place for end-of-life care (PPOC) (**Figure 1**). Univariant test showed that choosing home in outpatients (72.6%) was higher than in inpatients (59.7%), which shows a significant difference (P = 0.005). Moreover, the percentage of choosing home in women (73.4%) was higher than in men (62.2%) (P = 0.007). People with academic education (77.6%) were more likely to receive care at home than other people with elementary education (59.6%) and high school (74.4%) (P = 0.000). Additionally, Persians (76%) picked their homes more often than other races (P = 0.013). The logistic model showed that inpatient patients had a lower likelihood of selecting home than outpatient patients (OR:.613 (.383,.982), P =.042). Patients with academic education had a higher likelihood of choosing home than patients with elementary education, although this difference was not statistically significant (OR: 2.61 (1.29, 5.24), P =.007). Patients with high school education had similar chances of choosing home as patients with elementary education. Chances of choosing home in two groups of patients with mediate income (OR: 3.27 (1.49, 7.14), P = .003) and low (OR: 3.38 (1.52, 7.52), P = .003) were significantly higher than in patients with high income. In other words, by decreasing income, the chance of choosing the home increased, and patients with breast cancer had a better chance of choosing a home than with gastrointestinal cancer (OR: 1.70 (1.01, 2.89), P = .049) (**Table 2**).

**Table 2:** Correlations between variables and the preferred place of end-of-life care in cancer patients.

					Multiple logistic model	
Variable	Categories	HospitalN (%)	HomeN (%)	P-value	*OR (**CI 95%)	P-value
**Type of care**	Outpatient	118 (27.4%)	313 (72.6%)	0.005	Base Category	
	Inpatient	52 (40.3%)	77 (59.7%)		.613 (.383,.982)	.042
**Sex**	Male	71 (37.8%)	117 (62.2%)	0.007		
	Female	99 (26.6%)	273 (73.4%)			
**Age**	18-40	43 (28.1%)	110 (71.9%)	0.084		
	40-60	76 (27.8%)	197 (72.2%)			
	>60	51 (38.1%)	83 (61.9%)			
**Marital status**	single	39 (31.2%)	86 (68.8%)	0.78		
	married	130 (30.0%)	304 (70%)			
**Number of children**	0	18 (26.9%)	49 (73.1%)	0.19		
	1-3	102 (28.1%)	261 (71.9%)			
	≥4	42 (36.5%)	73 (63.5%)			
**Education**	Elementary	74 (40.4%)	109 (59.6%)	0.000	Base Category	
	High school	68 (25.6%)	198 (74.4%)		1.51 (.907, 2.51)	.113
	academic	22 (22.4%)	76 (77.6%)		2.61 (1.29, 5.24)	.007
**Jobs**	Employed	40 (31.7%)	86 (68.3%)	0.74		
	Unemployed	129 (30.2%)	298 (69.8%)			
**Ethnicity**	Fars	63 (24%)	199 (76%)	0.013		
	Lor	15 (28.8%)	37 (71.2%)			
	Tork	52 (34.9%)	97 (65.1%)			
	Kord	31 (39.7%)	47 (60.3%)			
	other	8 (50%)	8 (50%)			
**Income**	High	24 (57.1%)	18 (42.9%)	0.003	Base Category	
	Mediate	90 (29.6%)	214 (70.4%)		3.27 (1.49, 7.14)	.003
	low	54 (26.7%)	148 (73.3%)		3.38 (1.52, 7.52)	.003
**Type of cancer**	Gastrointestinal	52 (36.1%)	92 (63.9%)	0.006	Base Category	
	Breast	47 (21.6%)	171 (78.4%)		1.70 (1.01, 2.89)	.049
	Blood	23 (39.7%)	35 (60.3%)		.65 (.33, 1.29)	.225
	other	25 (30.1%)	58 (69.9%)		1.32 (.71, 2.46)	.381
**Insurance**	***Social security	50 (23.5%)	163 (76.5%)	0.006		
	****military	63 (31.3%)	138 (68.7%)			
	other	56 (39.2%)	87 (60.8%)			
**Time to diagnostic disease (month)**	≤6	55 (34%)	107 (66%)	0.14		
	>6	104 (27.7%)	271 (72.3%)			

*OR, odds ratio; **CI, confidence interval.

***Funder: Social Security Organization.

**Funder: Military Organization.

### Preferred place of death

Four hundred fifteen (75.2%) patients chose home as their PPOD, while 137 (24.8%) people chose the hospital (**Figure 1**). Choosing a home as a PPOD in married patients (77.6%) compared to single patients (66.7%) was higher (P = 0.014). Also, patients with Armed Forces Insurance with 82.1% were the most patients who chose home as the PPOD (P = 0.011). Moreover, patients with a disease period of less than 6 months (84.9%) compared to patients with a period of more than 6 months (72%) had chosen home as the PPOD (P = 0.002). However, the logistic model showed that married people have a higher chance of choosing the home than single people (OR: 1.62 (1.02, 2.57), P = .039). Besides, people with a disease period longer than 6 months had a significantly lower chance of choosing the home than people with a disease period of less than 6 months (OR:.468 (.286,.765), P = .002). (**Table 3**).

**Table 3:** Correlations between variables and the preferred place of death of cancer patients.

					Multiple logistic model	
Variable	Categories	HospitalN (%)	HomeN (%)	P value	OR (CI 95%)	P-value
**Type of care**	Outpatient	109 (25.7%)	315 (74.3%)	0.379		
	Inpatient	28 (21.9%)	100 (78.1%)			
**Sex**	Male	41 (22.7%)	140 (77.3%)	0.41		
	Female	96 (25.9%)	275 (74.1%)			
**Age**	18-40	45 (30.2%)	104 (69.8%)	0.13		
	40-60	58 (21.4%)	213 (78.6%)			
	>60	34 (25.8%)	98 (74.2%)			
**Marital status**	Single	41 (33.3%)	82 (66.7%)	0.014	Base category	
	Married	96 (22.4%)	332 (77.6%)		1.62 (1.02, 2.57)	.039
**Number of children**	0	16 (24.6%)	49 (75.4%)	0.67		
	1-3	93 (25.8%)	268 (74.2%)			
	≥4	24 (21.6%)	87 (78.4%)			
**Education**	Elementary	43 (23.8%)	138 (76.2%)	0.65		
	High school	65 (24.7%)	198 (75.3%)			
	academic	25 (26.3%)	70 (73.7%)			
**Jobs**	Employed	26 (21.1%)	97 (78.9%)	0.27		
	Unemployed	110 (25.9%)	314 (74.1%)			
**Ethnicity**	Fars	56 (21.8%)	201 (78.2%)	0.43		
	Lor	11 (21.6%)	40 (78.4%)			
	Tork	44 (29.9%)	103 (70.1%)			
	Kord	21 (26.9%)	57 (73.1%)			
	other	4 (25%)	12 (75%)			
**Income**	High	12 (28.6)	30 (71.4%)	0.92		
	Mediate	73 (24.3)	228 (75.7%)			
	low	51 (25.5)	149 (74.5%)			
**Type of cancer**	Gastrointestinal	29 (20.4)	113 (79.6%)	0.28		
	Breast	63 (28.9)	155 (71.1%)			
	Blood	14 (24.6)	43 (75.4%)			
	other	18 (22)	64 (78%)			
**Insurance**	Social security	57 (27)	154 (73%)	0.011		
	military	35 (17.9)	161 (82.1%)			
	other	45 (31.5)	98 (68.5%)			
Time to diagnostic disease (**month)**	≤6	24 (15.1)	135 (84.9%)	0.002	Base category	
	>6	104 (28)	268 (72%)		.468 (.286,.765)	.002

OR, odds ratio; CI, confidence interval.

## Discussion

In Iran, there are no official statistics on the PPOEOLC and the PPOD in cancer patients. Therefore, the purpose of this study was to determine the PPOEOLC and PPOD in cancer patients. Given that there is a view in Iranian culture that discussing death and dying with patients is inappropriate because of stress and poor patient morale, the paucity of study in this field is likely a result of cultural and religious constraints. Thus, preference about the place of death and place of end-of-life care is not a stable concept and can change over time through discussion between healthcare professionals and patients according to social, supportive, and individual conditions (32, 33).

By reviewing the literature, various studies have discussed the PPOEOLC and PPOD together (11, 12, 15, 34). However, in the present study, both concepts (PPOEOLC and PPOD) were surveyed using two separate questions. In the present study, most cancer patients chose home as their PPOEOLC and PPOD.

This result is consistent with a recent systematic review study by Fereidouni et al. (3), Choi et al. in South Korea (34), Yamagishi et al. in Japan (15), Skorstengaard et al. in Denmark (11), Alsirafy et al. in Egypt (35), Gu et al. in China (36), Lee et al. in Taiwan (37), and Nakamura et al. in Japan (38). A study by Brogaard et al. found that cancer patients chose 84% of their PPOEOLC, and 71% of their PPOD at home (12). Another systematic review shows that the home is the PPOD for most cancer patients worldwide (3). The reason why individuals in various nations choose to pass away at home is probably impacted by a number of things. For instance, patients who preferred to pass away at home were more likely to do so if they were less educated, lived with their spouse or family, or were from rural regions (34, 36). Some patients may have a history of adversity in their lives, which influences their decision (39).

Dying and caring at home may have religious significance, because the home environment can facilitate cultural and religious ceremonies at the end of life as an integral part of peaceful death (16). The cultural family-centered principle of Iranian-Islamic society and patients’ desire for family members to be present in bed when receiving end-of-life care is a feature of Iranian society that is effective to achieve the present result. According to other research, the capacity to address the patient’s fundamental requirements, patient privacy, a more soothing environment for the patient and caregiver, and simple access to home care support systems are the most prevalent reasons patients opt to get their care at home (16, 40, 41). The hospital was the second priority as the PPOEOLC and PPOD of cancer patients. Consistent with the present study, the study by Choi et al. in South Korea (34), Skorstengaard et al. in Denmark (11), and three other studies show that the hospital is the second priority as the PPOD and PPOEOLC (34, 36, 42, 43). The most common reasons of patients in choosing the hospital as the second PPOD and PPOEOLC in other studies include patients not wishing to be a burden on their family (40), having a reliable relationship between caregivers and patients (44), having a safer care environment, adequate facilities, and equipment to facilitate quality care, especially time to overcome pain and discomfort (45), poor functional status of the patient and the family’s inability to provide effective and quality care, and lack of the participation of the palliative care team at home (16).

In accordance with Iranian society’s culture, hospitals are seen as a secure and suitable setting for managing illness symptoms and suffering, particularly when doing so is challenging. The answer to the question about the PPOEOLC and PPOD was limited to two options of home and hospital. Other studies used places such as nursing home and hospice but in terms of the lack of development of hospice centers in Iran, as in many Middle-eastern countries (35), However, in countries where there are hospices, patients still choose home as their PPOD (9, 13, 46).

The findings of the current research are consistent with those of the studies by Jeurkar et al. (13) and Gu et al. (14), which found that married patients were more likely than single patients to choose home as their PPOD (36). This is most likely because the patient and spouse have a stronger emotional bond, which makes the home environment more calming for the patient. The results of the present study showed that outpatients had a significantly higher chance of choosing the home as a PPOEOLC than inpatients. Moreover, patients with a time to diagnostic disease the less than 6 months had a significantly higher chance of choosing the home as a PPOD than the patients’ time to diagnostic disease of more than 6 months. The result of this study is contrary to the study of Gu et al. (36). The reason for this difference is most likely the individual’s incompatibility with the hospital environment at the beginning of the illness. Inpatients and people who have been ill for a long time are more likely to adapt to the hospital environment due to the longer hospital stay.

The patients with academic level of education are more likely to choose home as the PPOEOLC than patients with elementary and high school levels of education. This result is inconsistent with the study of Choi et al. (34) and the study of Chen et al. (47). The difference may be explained by the fact that patients with greater levels of education are more aware of and knowledgeable about conditions for self-care at home, which enhances the possibility that they will choose the home. In our study, middle- and lower-income people chose home as their PPOEOLC more than high-income people; the reason for this choice is probably due to the financial inability of these people to pay for care and equipment in the hospital.

According to World Health Organization’s World Cancer Statistics in 2020, the most common type of cancer in 2022 is breast cancer (2.26 million cases) (4). In our study, the type of disease was also a significant factor in choosing the home as a PPOEOLC, so that people with breast cancer chose home more than people with other cancers. The result of our study was in line with the study of Chen and the study of Blanchard et al. In the Blanchard study, breast cancer patients also chose the home as the PPOD (47, 48).

## Conclusion

The results of the present study show that the majority of Iranian cancer patients chose home as the PPOEOLC and the PPOD. According to the findings of this study, experts advocate paying more attention to the preferences of terminally ill patients, strengthening the system of home healthcare. This will be accomplished through enhancing the home care system *via* the development of suitable infrastructure, insurance coverage, the drafting of executive directives, and the incorporation of palliative care into home care services. In addition, emphasizing death at home requires a fair distribution of health resources. For this purpose, more resources need to be allocated to home health care.

### The strength of the study

The strength of the present study lies in the fact that we used patients’ statements about their preferences rather than a proxy statement made by others, such as a family caregiver or other caregivers. Another strength of this study is that the question is asked from cancer patients about the PPOEOLC and PPOD, not from the general population without diagnosis; in fact, when people are well and live without diagnosis of life-limiting diseases or do not face death, they may use an abstract and unrealistic answer, while in our study this answer was concrete.

### Limitations

Despite the inclusion of a wide range of potential predictors, observational studies may never be able to reduce the effect of confounding variables to zero. As patients approach their last days of life, cross-sectional evaluations may also alter the dynamic decision-making process of priorities, predictors of death, and home care. Another limitation of this study is that the “no preference” option was not considered as an answer that is suggested to be considered in future studies.

### Implications for practice and future research

To achieve the patient’s preferences at the end of life, it is important to have a preferred place to discuss death and document the decision. Therefore, it is important to ensure that all patients have the opportunity to speak about this issue in a supportive, practical, and compassionate manner. To increase their competency and confidence in end-of-life conversations, medical and nursing professionals’ training may be strengthened. Death in hospitals is anticipated to predominate in the future, despite minor variations in the location of death throughout time. Therefore, it is necessary to take measures in this regard to improve the experiences of patients and their families at this time. Further research is needed on the impact of deprivation and other socioeconomic factors on preferences, the reasons for lack of discussing the place of death, and the lack of expression of preference or change of preference.

## Data availability statement

The raw data supporting the conclusions of this article will be made available by the authors, without undue reservation.

## Author contributions

Study design: AF, MR, and SB; data collection: FH, MJ, MK, and ME; data analysis: MS and SB; study supervision: SB; manuscript writing: AF, MR, MS, FH, MK, MJ, ME, and SB; critical revisions for important intellectual content: AF, SB, and MR. All authors contributed to the article and approved the submitted version.

## Acknowledgments

Thanks to guidance and advice from “Clinical Research Development Unit of Baqiyatallah Hospital.”

## Conflict of interest

The authors declare that the research was conducted in the absence of any commercial or financial relationships that could be construed as a potential conflict of interest.

## Publisher’s note

All claims expressed in this article are solely those of the authors and do not necessarily represent those of their affiliated organizations, or those of the publisher, the editors and the reviewers. Any product that may be evaluated in this article, or claim that may be made by its manufacturer, is not guaranteed or endorsed by the publisher.
